# A case report of renal cell carcinoma metastasis revealed through late‐onset thyroid nodules

**DOI:** 10.1002/cnr2.2113

**Published:** 2024-06-21

**Authors:** Abolfazl Khalafi‐Nezhad, Ali Zamani, Mahya Amini, Shahrzad Negahban

**Affiliations:** ^1^ Hematology Research Center, Department of Hematology, Medical Oncology and Stem Cell Transplantation Shiraz University of Medical Sciences Shiraz Iran; ^2^ Associate Professor of Endocrinology & Metabolism Department of Internal Medicine, Endocrine and Metabolism Research Center Shiraz University of Medical Sciences Shiraz Iran; ^3^ Department of Internal Medicine Shiraz University of Medical Sciences Shiraz Iran; ^4^ Department of pathology and cytology Dr. Daneshbod Pathology Laboratory Shiraz Iran

**Keywords:** dormancy phenomenon, metastasis, renal cell carcinoma, thyroid nodules, thyroidectomy

## Abstract

**Background:**

Renal cell carcinoma (RCC) is one of the most common and prevalent cancers all around the world with a prevalence of 3%. Approximately twenty percent of patients present with metastasis at the time of diagnosis, while late metastasis in renal cell carcinoma is a quite familiar phenomenon. Head and neck and particularly thyroid metastasis from RCC are rare events.

**Case:**

We present a case of a 75‐year‐old woman who developed thyroid nodules 13 years after nephrectomy for RCC. Diagnosis confirmed metastatic RCC through clinical history, histomorphology, and immunohistochemistry. Imaging studies revealed thyroid lesions without metastasis in other organs. The patient underwent total thyroidectomy and remains symptom‐free after 2 years of follow‐up.

**Conclusion:**

This case highlights the importance of considering metastatic lesions is crucial in managing thyroid nodules in patients with a history of cancer, particularly RCC.

## 1 INTRODUCTION

Renal cell carcinoma (RCC) is the predominant form of kidney neoplasms and accounts for approximately 3% of all malignant tumors in humans.^1^ Survival is highly dependent on the stage of disease at diagnosis, with a 5‐year survival of 93% for stage 1, 72.5% for stage 2 and 3 regional disease, and only 12% for stage 4.^2^

Risk stratification in RCC plays a crucial role in determining prognosis, treatment decisions, and follow‐up strategies. Several established risk stratification systems are commonly used in clinical practice. Tailored follow‐up regimens are crucial in the management of high‐risk RCC, considering the potential for disease recurrence and the emergence of new insights regarding adjuvant therapies. immune checkpoint inhibitors, such as nivolumab and pembrolizumab, have also shown promising results in the adjuvant setting for high‐risk RCC. These agents enhance the immune response against cancer cells and have the potential to improve outcomes in this patient population.^3^

Roughly around 30%–50% of patients have metastasis at the time of diagnosis. Although metastasis to bone, brain, and lungs is common in RCC, it may spring up in unusual sites such as skin, testis, and maxillary.^2^, ^4^ Most recurrences, approximately 78%, occur within the first five years following nephrectomy. However, late metastasis, defined as distant recurrence occurring more than 10 years after nephrectomy, is not an uncommon occurrence in patients with renal cell carcinoma.^5^ The most common sites for metastatic RCC are the lungs, lymph nodes, bone, and liver. The precise mechanism of RCC metastasis is still unknown; however, hematogenous metastasis, lymphogenous dissemination, and direct invasion are some possible ways.^6^, ^7^

Metastasectomy is typically reserved for cases where complete resection is feasible unless the primary goal of treatment is palliative symptom management. When considering surgery, it is crucial to weigh the potential morbidity and mortality associated with metastasectomy.^8^, ^9^

In addition to surgery, targeted therapies such as tyrosine kinase inhibitors (TKIs) and immune checkpoint inhibitors (ICIs) have emerged as notable alternatives for treating metastatic renal cell carcinoma (RCC). These systemic therapies have demonstrated impressive effectiveness in controlling tumor growth and improving survival rates in patients with metastatic disease. Furthermore, there are less invasive treatment options available for metastatic RCC patients. Procedures like radiofrequency ablation (RFA), stereotactic ablative radiotherapy (SABR), and cryoablation (CA) offer alternative approaches that are less invasive compared to surgical interventions. These techniques can be utilized to destroy or ablate tumors while minimizing the impact on surrounding healthy tissues. When determining the most suitable treatment approach, it is crucial to consider various factors such as the feasibility of complete resection, the potential for palliative symptom relief, morbidity and mortality associated with surgery, and the effectiveness of targeted therapies or alternative interventions. A comprehensive evaluation by a multidisciplinary team is essential to ensure that patients receive the most appropriate and effective treatment for their metastatic RCC.^9^

Even though the thyroid gland has an abundant blood supply, metastatic thyroid cancer is rare, 1.15%–3%. Faster blood flow, high oxygen saturation, and high iodine content of the thyroid gland are proposed as the impeding factors for the growth of metastasis. Metastasis to the thyroid is not uncommon in biopsy; the incidence is 1.2%–24%. The most common primary malignant tumor metastasizing to the thyroid gland is RCC followed by colorectal carcinoma, pulmonary, and breast carcinoma.^10^, ^11^, ^12^

The uniqueness of this case lies in the rare occurrence of RCC metastasis to the thyroid gland, the significant time interval between the initial nephrectomy and the detection of metastasis, and the successful management through total thyroidectomy. These findings contribute to the medical literature on the behavior and clinical presentation of renal cell carcinoma and highlight the importance of considering metastasis to the head and neck region in patients with a history of RCC.

## 2 CASE

The 75‐year‐old female patient presented to her endocrinologist at Namazi Hospital with a complaint of a neck mass in April 2021. She denied experiencing any difficulty in breathing (dyspnea), changes in voice (hoarseness), or difficulty swallowing (dysphagia). Thirteen years ago, the individual underwent a radical nephrectomy to treat a tumor in their right kidney. A histopathological study of the surgically removed tissue confirmed that the tumor was clear cell renal cell carcinoma (pT1a N0 M0). The patient has been undergoing annual follow‐up with abdominal CT and chest X‐ray for 5 years following radical nephrectomy, along with annual history and physical examinations up to the present time.

During the physical examination, multiple nodules were identified in both thyroid gland lobes. The results of the thyroid function test showed a thyroid‐stimulating hormone (TSH) level of 1.08 mIU/L (normal range: 0.4–4.0 mIU/L), a free thyroxine (FT4) level of 1.6 ng/dL (normal range: 0.7–1.8 ng/dL), and a free triiodothyronine (FT3) level of 2.2 pg/mL (reference range: 2.3–4.2 pg/mL).

An ultrasonographic examination was performed, revealing the presence of multiple nodules in both lobes. The largest nodule, measuring 22 × 21 mm, was found in the left lobe and appeared hypoechoic and solid. Another smaller nodule with an irregular border and peripheral calcification was observed in the right lobe.

**FIGURE 1:**
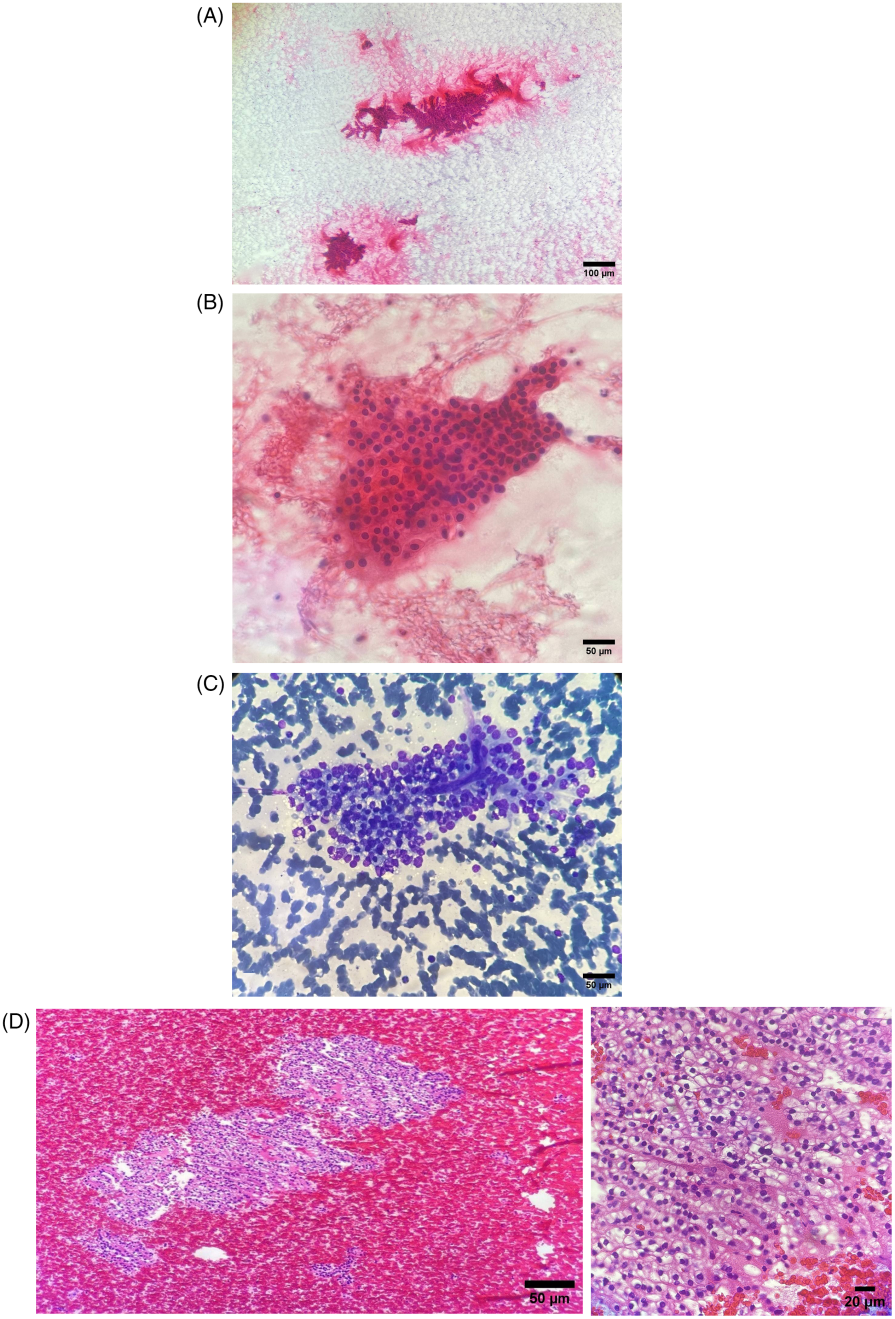
Fine needle aspiration biopsy of a thyroid nodule. (A, B) Papillary clusters and three‐dimensional arrangements of abnormal cells observed (Papanicolaou stain; magnification, ×100 and ×200; scale bar, 100 and 50 μm). (C) Presence of a fibrovascular core and vacuolated cytoplasm with clear appearance (Wright‐Giemsa stain; magnification, ×200; scale bar, 50 μm). (D) Cellblock analysis reveals distinct well‐defined cells with clear cytoplasm (H&E stain; magnification, ×200 and ×400; scale bar, 50 and 20 μm). No colloid material is detected in the smears or cell block.

A fine needle aspiration (FNA) procedure was conducted to investigate the left thyroid lobe nodule further. The aspirated nodule yielded highly cellular smears, which displayed three‐dimensional clusters, papillae, and sheets of neoplastic cells. These cells exhibited round and ovoid hyperchromatic nuclei, conspicuous nucleoli, and abundant clear and finely granular cytoplasm. The background of the smears appeared bloody. (Figure 1).

**FIGURE 2:**
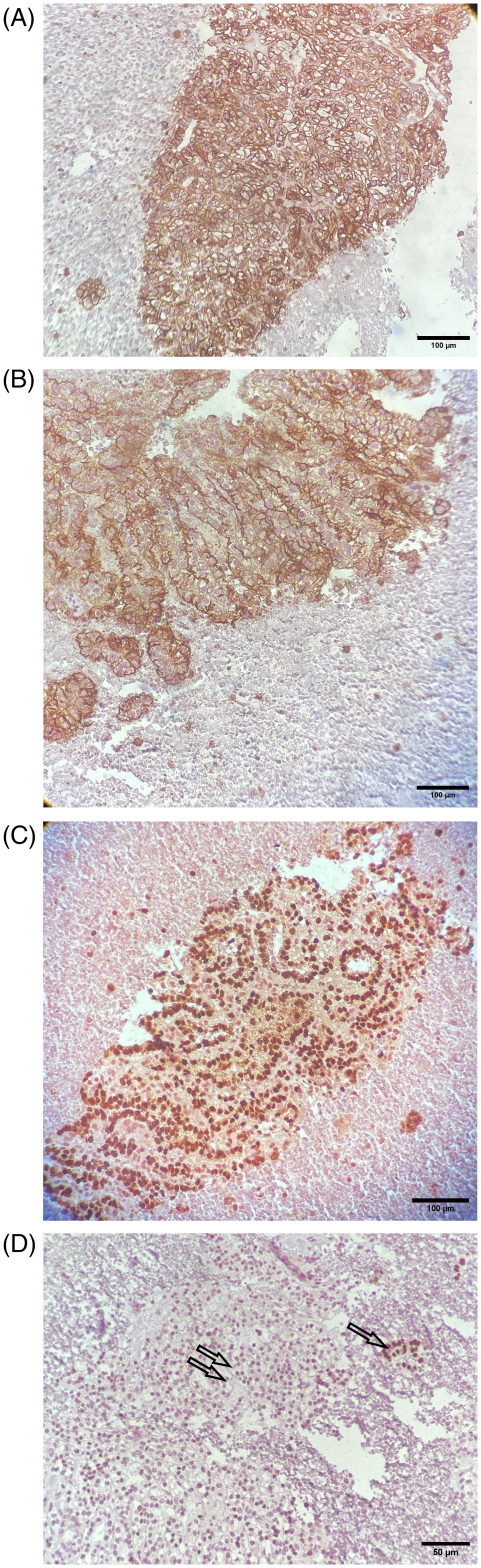
Immunohistochemical staining of a metastatic lesion in the thyroid. Expression patterns of tumor cells: (A) Carbonic anhydrase IX (CA IX) shows diffuse box‐like membrane staining in the tumor cells (magnification, ×200; scale bar, 100 μm). (B) CD10 displays apical membrane staining (magnification, ×200; scale bar, 100 μm). (C) Tumoral cells demonstrate diffuse nuclear staining (magnification, ×200; scale bar, 100 μm). (D) Tumoral cells show no staining for PAX8 (indicated by two arrows), while a few preserved follicles exhibit nuclear staining (indicated by one arrow) (magnification, ×400; scale bar, 50 μm).

An immunohistochemistry (IHC) study on cell block was performed. Tumoral cells showed positive staining for CD10, PAX8 and CA‐IX and they were negative for TTF1 (Figure 2).

**FIGURE 3:**
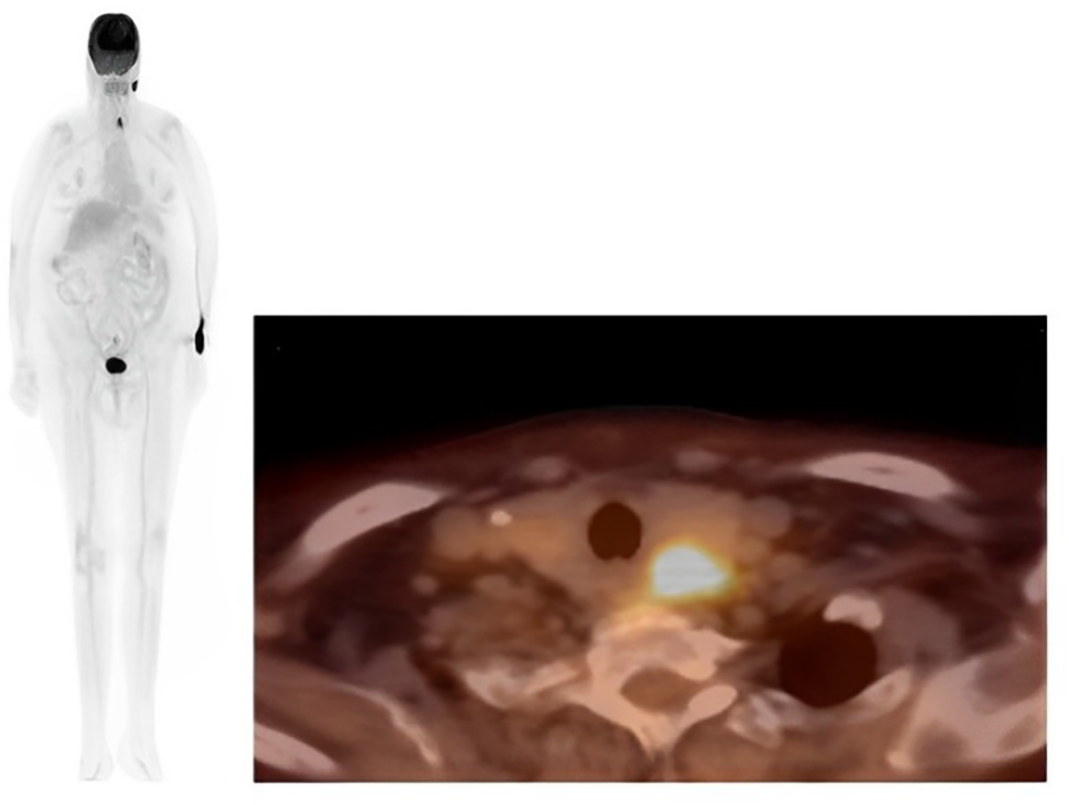
A PET/CT scan of a patient revealing a fused pathological uptake in the thyroid gland, suggesting metastatic involvement in a patient diagnosed with renal cell carcinoma.

Consequently, based on the patient's clinical history, histomorphology, and immunohistochemistry (IHC) findings (Figure 2), a diagnosis of metastatic renal cell carcinoma (RCC) was established. To assess the potential presence of metastasis in other organs, a fluorodeoxyglucose (FDG) PET‐CT scan was conducted. The scan revealed a low attenuation lesion in the lower aspect of the left thyroid lobe, indicating FDG uptake, and another non‐FDG‐avid low attenuation lesion in the right thyroid lobe, which contained an eccentric calcified focus (Figure 3). No evidence of metastasis was detected in other areas of the body.

In light of these findings, a successful total thyroidectomy was performed without any complications during or after the operation.

Based on the histopathology report, the examination of the thyroid sample revealed a neoplastic nodule measuring 23 × 22 mm in the left lobe. The nodule consisted of large clear cells. Immunohistochemical staining demonstrated that the tumor cells were positive for vimentin, CD10, PAX8, and CA‐IX. However, they tested negative for thyroglobulin, thyroid transcription factor‐1 (TTF‐1), and chromogranin. These findings confirmed the renal origin of the tumor, indicating clear cell carcinoma.

The patient has been regularly followed up for 2 years, and there have been no complaints or signs of recurrence or metastasis.

## 3 DISCUSSION

The findings of this case study contribute to the existing literature by highlighting the rare occurrence of late metastasis to the thyroid gland from renal cell carcinoma (RCC) many years after nephrectomy. Isolated thyroid metastasis is uncommon, and the diagnosis was confirmed through clinical history, histomorphology, and immunohistochemistry. While metastasis to the thyroid gland is relatively rare, RCC is among the most frequently reported malignancies to metastasize to the thyroid gland, usually involving concurrent metastatic involvement of other organs. However, isolated thyroid metastasis is infrequent.^13^

The case report emphasizes the importance of utilizing ultrasound imaging, fine needle aspiration with immunohistochemical staining, and PET scans for accurate diagnosis and evaluation of metastatic lesions. In this particular case, no evidence of metastasis was detected in other areas of the body, and a successful total thyroidectomy was performed without complications.

Sonographically, metastatic RCC to the thyroid gland is characterized by solid or solid cystic, irregularly shaped nodules with heterogeneous echogenicity, with or without calcification.^14^ Although ultrasound imaging alone may not provide definitive discrimination, fine needle aspiration with immunohistochemical (IHC) staining can help differentiate primary thyroid carcinoma from metastatic thyroid lesions, particularly in patients with a known history of malignancy. Additionally, PET scans are considered beneficial complementary tools for evaluating distant metastasis and guiding treatment planning.^15^

Renal cell carcinoma is known for synchronous and metachronous metastasis.^16^ About 30% of patients with localized RCC experience recurrence after surgery, with the majority occurring within 5 years of resection.^17^ However, 4%–11% of RCC patients may experience relapse even after 10 years.^18^, ^20^ Our patient encountered this complication 13 years after the initial successful therapy. Late metastasis cases, including those with disease‐free survival exceeding 10 years, have been reported.^19^, ^20^

The concept of temporary mitotic arrest or cellular dormancy may partially explain the occurrence of overt metastasis or recurrence many years after successful treatment of the primary tumor. Dormancy refers to a reversible nonproliferating state or cell cycle arrest in G0. RCC and cutaneous malignant melanoma are among the most common malignancies associated with this unique feature. RCC can remain in a dormant state for decades. The underlying mechanisms responsible for reversing mitotic arrest and leading to clinically detectable metastatic lesions in different organs are poorly understood. Aging‐related decline in immune function may influence host‐tumor interaction, promoting the proliferation of dormant malignant cells in various tissues.^20^, ^21^, ^22^

Metastasis to the head and neck, particularly the thyroid gland, is uncommon. However, recent studies have revealed that approximately 1.9%–22.4% of patients with disseminated metastasis have thyroid gland metastasis based on autopsy results.^10^, ^11^ On the other hand, thyroid nodules are a common problem in adults, and their incidence increases with age. However, less than 5% of these nodules are malignant.^23^, ^24^ It has been proposed that the thyroid gland's high vascularity and iodine content are significant factors impeding tumor implantation. However, any structural deformity in the thyroid gland may increase the chance of tumor seeding.^13^, ^24^

## 4 CONCLUSION

This case highlights the importance of considering metastatic RCC in the diagnosis of thyroid nodules, especially in patients with a history of RCC. Ultrasonography, fine needle aspiration with immunohistochemistry, and PET scans aid in diagnosing and evaluating metastatic lesions. The presence of thyroid nodules in a patient with a history of RCC requires a change in approach for evaluating and managing the nodules. Ultrasound alone may not discriminate between primary thyroid carcinoma and metastatic lesions. Therefore, FNA with immunohistochemical staining aids differentiation in patients with a known history of malignancy. Further research is needed to understand the mechanisms of late metastasis and factors contributing to tumor seeding in the thyroid gland. In summary, a comprehensive approach involving FNA with immunohistochemical staining, ultrasound, PET scans, and consideration of metastatic lesions is crucial in managing thyroid nodules in patients with a history of cancer, particularly RCC.

## AUTHOR CONTRIBUTIONS

**Abolfazl Khalafi‐Nezhad:** Conceptualization; writing – original draft; investigation; methodology; supervision; writing – review and editing; project administration. **Ali Zamani:** Conceptualization; supervision; methodology; validation; writing – original draft; writing – review and editing. **Mahya Amini:** Conceptualization; data curation; formal analysis; investigation; methodology; software; writing – original draft. **Shahrzad Negahban:** Investigation; data curation; methodology; validation; writing – original draft.

## CONFLICT OF INTEREST STATEMENT

The authors have stated explicitly that there are no conflicts of interest in connection with this article.

## ETHICS STATEMENT

The current study was approved by the Ethical Committee of Shiraz University of Medical Sciences. We affirm that explicit consent for publication in both print and electronic formats has been obtained from the patient.

## Data Availability

The data that support the findings of this study are available on request from the corresponding author. The data are not publicly available due to privacy or ethical restrictions.
